# Differences in the Stool Metabolome between Vegans and Omnivores: Analyzing the NIST Stool Reference Material

**DOI:** 10.3390/metabo13080921

**Published:** 2023-08-07

**Authors:** Raquel Cumeras, Tong Shen, Luis Valdiviez, Zakery Tippins, Bennett D. Haffner, Oliver Fiehn

**Affiliations:** 1 West Coast Metabolomics Center, University of California Davis, Davis, CA 95616, USA; raquel.cumeras@urv.cat (R.C.);; 2 Oncology Department, Institut d’Investigació Sanitària Pere Virgili (IISPV), Univeristat Rovira i Virgili (URV), 43204 Reus, Spain; 3 Nutrition and Metabolism Department, Institut d’Investigació Sanitària Pere Virgili (IISPV), Univeristat Rovira i Virgili (URV), 43204 Reus, Spain

**Keywords:** reference material, stool, metabolomics, diet, omnivore, vegan, mass spectrometry

## Abstract

To gain confidence in results of omic-data acquisitions, methods must be benchmarked using validated quality control materials. We report data combining both untargeted and targeted metabolomics assays for the analysis of four new human fecal reference materials developed by the U.S. National Institute of Standards and Technologies (NIST) for metagenomics and metabolomics measurements. These reference grade test materials (RGTM) were established by NIST based on two different diets and two different samples treatments, as follows: firstly, homogenized fecal matter from subjects eating vegan diets, stored and submitted in either lyophilized (RGTM 10162) or aqueous form (RGTM 10171); secondly, homogenized fecal matter from subjects eating omnivore diets, stored and submitted in either lyophilized (RGTM 10172) or aqueous form (RGTM 10173). We used four untargeted metabolomics assays (lipidomics, primary metabolites, biogenic amines and polyphenols) and one targeted assay on bile acids. A total of 3563 compounds were annotated by mass spectrometry, including 353 compounds that were annotated in more than one assay. Almost half of all compounds were annotated using hydrophilic interaction chromatography/accurate mass spectrometry, followed by the lipidomics and the polyphenol assays. In total, 910 metabolites were found in at least 4-fold different levels in fecal matter from vegans versus omnivores, specifically for peptides, amino acids and lipids. In comparison, only 251 compounds showed 4-fold differences between lyophilized and aqueous fecal samples, including DG O-34:0 and methionine sulfoxide. A range of diet-specific metabolites were identified to be significantly different between vegans and omnivores, exemplified by citrinin and C17:0-acylcarnitine for omnivores, and curcumin and lenticin for vegans. Bioactive molecules like acyl alpha-hydroxy-fatty acids (AAHFA) were differentially regulated in vegan versus omnivore fecal materials, highlighting the importance of diet-specific reference materials for dietary biomarker studies.

## 1. Introduction

Microbial metabolites in human and mammalian feces link gut microbiota compositions to metabolic biomarkers of health and disease [1,2]. Numerous factors determine the diversity of the microbiota in humans, with diet being a key contributor [3]. Specific dietary components act as substrates for microbial metabolism, shaping microbiome composition and function. While metagenomics sequencing of microbial communities has been standardized, metabolomic characterization of stool currently lacks standards. Here, samples with certified values or consensus estimates are used to develop and benchmark analytical workflows for community-wide comparisons of metabolomics results. For example, the U.S. National Institute of Standards and Technologies (NIST) standard reference material 1950 (Metabolites in Frozen Human Plasma) is used in many clinical trials and round-robin tests as validation standard [4].

To close the gap for certified stool materials, the Gut Microbiome Committee of the Institute for the Advancement of Food and Nutrition Sciences (IAFNS; formerly the International Life Sciences Institute) aims to identify and eventually quantify gut microbial metabolites that have been linked to diet and health [5]. NIST and IAFNS are now collaborating to develop a suite of human whole stool reference materials (RMs) to validate both metagenomics measurements associated with Fecal Microbiome Transplants (FMTs) and other live biotherapeutic products as well as metabolomic measurements to identify new biomarkers associated with the health of the human gut microbiome. Four pooled whole stool prototype research grade test materials (RGTM) collected from vegans and omnivores have been developed by NIST [6,7] for use in method harmonization and quality control (QC) for next-generation sequence (NGS) metagenomics and MS- and NMR-based untargeted metabolomics. These RGTMs have been demonstrated to be homogeneous with respect to both the microbial taxa and key metabolites. They are being evaluated for longer-term (>6 months) stability in addition to any differences in the aqueous and lyophilized storage conditions. They are being used as test materials in an ongoing gut microbiome metabolomics NIST interlaboratory study [6].

Here, we present a multiplatform metabolomics analysis of four NIST fecal RGTM of fecal matter, which are representative of diet (vegan/omnivore) and sample treatment (lyophilized/aqueous). Using six metabolomics assays with three different mass spectrometry platforms, we demonstrate both the diversity of stool metabolites and the large differences between vegan and omnivore fecal matter.

## 2. Materials and Methods

### 2.1. Samples

The NIST human fecal research grade reference materials used were as follows: RGTM 10162 vegan homogenized (lyophilized); RGTM 10171 vegan homogenized (aqueous); RGTM 10172 omnivore homogenized (lyophilized); and RGTM 10173 omnivore homogenized (aqueous). The different RGTM’s were stored at −80 °C before use. Ten technical replicates of each RGTM were analyzed in all cases. To ensure reproducibility and limit losses of metabolites, samples were vortexed, thawed in ice and vortexed before starting the extraction process. Samples were randomized in all untargeted methods (CSH-, HILIC-, PFP-accurate mass MS/MS and GC-TOF MS). A targeted LC-MS/MS method was used for bile acids. Pooled matrix QC samples were used for intra-study assessment, and blank samples were used for system suitability and influence of blank signal. QC samples were prepared by mixing equal amounts of each sample from all the groups, prepared identically to the analytical sample, and analyzed after each set of 10 sample runs.

### 2.2. Extraction

In all untargeted extraction procedures described below, method blanks were used and prepared with the exact same extraction, derivatization, and resuspension procedures per assay. Biphasic extraction (for lipidomics-, HILIC-, and GC-TOF mass spectrometry) was carried out as follows: To 100 uL of aqueous RGTM or 2.0 ± 0.25 mg of lyophilized RGTM, 975 μL ice-cold 3:10 (*v*/*v*) MeOH/MTBE solvent mixture was added. After extraction for 5 min at 4 °C, 188 μL water was added and centrifuged for phase separation. Then, 350 μL of the upper organic phase was dried down for lipidomic analysis (one is a backup), 110 μL of the lower phase was dried down for HILIC- and GC-TOF MS analysis. Back-up aliquots were used to create quality control pool samples. Polyphenol extraction: To 100 uL of aqueous RGTM or 2.0 +/− 0.25 mg of lyophilized RGTM, 1 mL cold 80:20 MeOH/H_2_O was added. After extraction for 5 min at 4 °C, 450 uL aliquots were dried and store at −20 °C until analysis. Bile acid extraction for lyophilized samples: 1.0 mg sample was extracted with 500 µL ice cold methanol that included 10 µL of a 250 nM bile acid internal standard mixture and 10 µL of 1 µM CUDA/PHAU internal standard mix. Each sample was ground by 2 × 3 mm stainless steel beads at 1500 rpm for 1 min and extracted twice with 500 µL 500µL ice-cold methanol each, then dried down and stored at −20 °C until analysis.

*Resuspension with internal standards:* All LC-MS resuspension solvents included the internal standard [12-[(cyclohexylamine) carbonyl]amino]-dodecanoic acid], CUDA), as system suitability quality control standard. *Lipidomic extracts* were resuspended in 100 uL of 9:1 *v*/*v* methanol–toluene including six deuterated internal standards (C_10_-, C_12_- and C_18_-acylcarnitines, C_20:4_-, C_18:1_- and C_16:0_- free fatty acids) plus C_17_ sphingosine and the Avanti Ultimate^®^ SPLASH kit. *HILIC-MS/MS extracts* were resuspended in 100 uL of 8:2 *v*/*v* acetonitrile–water including 19 deuterated internal standards (C_2_-acylcarnitine, N-methylhistamine, 1-methylnicotinamide, betaine, creatine, creatinine, ornithine, cystine, asparagine, phenylalanine, histidine, isoleucine, arginine, proline, threonine, glutamine, glutamic acid, valine, aspartic acid), plus the tripeptide Val-Tyr-Val. Polyphenol extracts were resuspended in 100 uL of 7% acetonitrile in water including 11 deuterated internal standards (hippuric acid, caffeine, epigallocatechin gallate, cinnamic acid, trans-resveratrol, daidzein, genistein, apigenin, 2-hydroxyfluorene, quercetin and resperpine). *Bile acids*: Samples were resuspended in 100 µL methanol with targeted internal standards, CUDA and PHAU (1-cyclohexyl urea 3-dodecanoic acid). Aliquots of hydrophilic extracts (for GC-TOF MS) were resuspended in 500 μL 3:3:2 (*v*/*v*/*v*) ACN:IPA:H_2_O (degassed) and dried again to remove remnants of proteins or lipids. Methoximation and trimethylsilylation derivatization solutions included C_08_- to C_30_- fatty acid methyl esters as retention index markers as published previously [8].

*Data acquisition*: All analyses included method blanks and quality controls prior to 10 randomized samples. All untargeted LC-MS/MS was performed on a Vanquish UPLC with a Q-Exactive HF mass spectrometer using top-4 data dependent acquisition with eight-fold iterative exclusion runs per RGTM. Chromatography conditions were used as published before for lipidomics [9], HILIC [9], GC-TOF MS [8] and bile acids using a Waters C_18_ BEH 100 mm × 2 μm column with a Sciex Qtrap 6500+ mass spectrometer [10].

### 2.3. Data Processing and Compounds Identification

All untargeted accurate mass MS/MS data were processed using MS-DIAL v4.60 [11] and curated via MS-FLO [12]. Processed data were curtailed via blank reduction (signal/noise > 3) and normalized to the sum of internal standards. Compound annotations were performed by combining NIST20 and the MassBank.us (MassBank of North America) libraries. Metabolites with relative standard deviations (%RSDs) higher than 30% in the pooled samples were excluded in all cases. Missing values were estimated using KNN. Annotation confidence is reported according to the levels proposed by the Metabolomics Standards Initiative (MSI). MSI 1 refers to MSMS, m/z and retention time matches; MSI 2 includes MS/MS and accurate m/z matches; MSI 3 is reported by m/z and retention time matches (constructed by authentic compounds or validated by retention time projection); and MSI 4 are unknowns (not reported here). Compound chemical classes were retrieved using the web-based application ClassyFire [13].

Untargeted GC-TOF MS data were processed using the BinBase algorithm [14] as published previously [8]. Bile acids targeted data were processed using Sciex MultiQuant version 3.0.2 peak integration and peak area computation for targeted bile acid profiling.

We also performed a GC-TOF MS assay for short chain fatty acids (acetate, propionate, butyrate and similar compounds) using tert.butyl-dimethylsilylation as derivatization. However, for the sample analyzed here, the obtained results were below the lowest calibration standard point curve and, hence, no data on this compound class can be reported.

### 2.4. Statistics

All untargeted data was merged in a unique table, containing 3927 entries. Targeted data was treated separately. MetaboAnalyst 5.0 [15] was used for the untargeted methods. First, an exploratory PCA was carried out to check for outliers. Statistical methods used are ANOVA and Partial Least Squares Discriminant Analysis (PLS-DA) (with a z-score scale). For the box-plot statistical plots, the open statistical platform JAMOVI [16] was used.

### 2.5. Compounds Metadata

In Supplementary Table S1, we provide a detailed list of all 3563 compounds with their common names, MW, chemical formula, major identifiers (InChIKey, SMILES, PubChem ID, HMDB, LIPIDMAPS, DrugBank, EPA DSSTox, ChEBI and BioCyc), and their chemical classes. The Chemical Translation Service [17] was used to retrieve the chemical compound information and identifiers, while for the chemical class, the ClassyFire web application [13] was used. A manual check was performed for the compounds that did not return a result. The heatmap was carried out with Ward clustering and Euclidean distance. Chemical classes were plotted using the open-source data visualization framework RAWGraphics [18].

## 3. Results and Discussion

### 3.1. Fecal Metabolome Database

**Figure 1:**
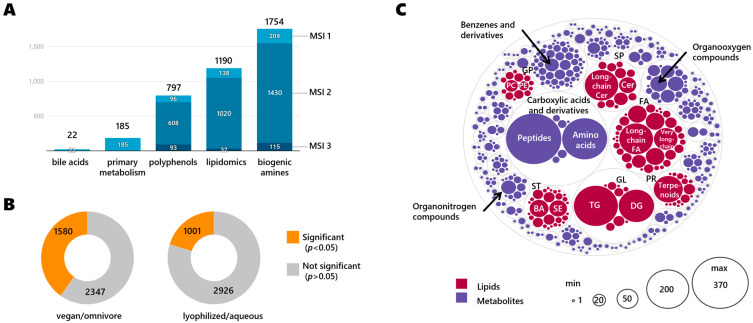
Overview of annotated compounds per assay and per chemical class. (**A**) Number and MSI level of annotated compounds found per assay combining all four RGTMs. (**B**) Significantly different compounds (two-sided t-test, raw *p* < 0.05) in study groups (**C**) Distribution of annotated compounds per chemical class. Chemical main classes indicated by grey lines, individual classes marked by colored circles. Lipid acronyms: FA: fatty acyls/acids; GL: glycerolipids; TG: triglycerides; DG: diglycerides; SP: sphingolipids; Cer: ceramides; PR: prenol lipids; ST: sterol lipids; BA: bile acids; SE: steryl esters; GP: glycerophospholipids; PC: phosphatidylcholines; PE: phosphatidylethanolamines.

With the multi-assay analysis performed, we annotated 3563 unique compounds (Table S1) via accurate mass and MS/MS matching. Each compound has been provided with relevant compound data (InChIKey, MW, chemical formula, SMILES), identifiers (Puchem CID, HMDB ID, KEGG ID, LIPIDMAPS ID, DrugBank ID, EPA DSSTox ID, ChEBI ID, BioCyc ID), and chemical classes. This has been provided to ensure the re-usability of the data in future studies. In total, 3210 annotated compounds were detected in a single assay, 328 compounds were found in 2 assays, 18 compounds were found in 3 assays, and 7 compounds were found in 4 assays (deoxycholic acid, lithocholic acid, FA 16:0 (palmitic acid), FA 18:1 (oleic acid), sphingosine, LPC 18:1 and LPC 16:0). A total of 22 bile acids were included in a targeted method (Table S2). For each RGTM we performed 10 randomized measurements to ensure statistical significance and to reduce the experimental bias. Figure 1A gives a summary of the number of annotated compounds per assay and their MSI levels. Bile acids (BA) are considered MSI 1 level, matching authentic standards, similar to primary metabolism compounds detected via GC-TOF MS because they were identified by the retention index-based mass spectral BinBase database that is supported by the Fiehnlib library of standards. Per assay, the following numbers of compounds were annotated: 1755 metabolites in the biogenic amines assay (HILIC-accurate mass MS/MS); 1190 lipids (CSH C18-accurate mass MS/MS); 797 polyphenols (PFP-accurate mass MS/MS); 185 primary metabolism (GC-TOF MS); and 22 bile acids (C18-QTRAP MS/MS).

By far, the most differences were found when comparing fecal matter from vegans versus omnivores, independent of the formulation (lyophilized or aqueous) in which the material was presented (Figure 1B). A total of 910 compounds were found with a fold-change equal to or higher than 4 (log2FC = 2), including 494 compounds with a fold-change higher than 8 (log2FC = 3). This data shows the high diversity and variation of fecal compounds between omnivores and vegan diets, with 96 compounds approaching absence and presence at fold-changes higher than 32 (log2FC = 5) (Figure S1). Figure 1C gives a classification overview of annotated compounds per chemical class. The most prevalent chemical main class was labeled by the ClassyFire software as ‘*Carboxylic acids and derivatives*’ with 653 metabolites, consisting mainly of classes of 371 peptides and 240 amino acids. The second most abundant chemical main class was ‘*fatty acyls*’ (FA) with 517 lipids, mainly 149 long-chain fatty acids and alcohols and 70 very-long-chain fatty acids. Then, per order of abundance, we annotated 439 *‘glycerolipids’* consisting of 236 triglycerides and 153 diglycerides; 283 *‘sphingolipids’* (SP) that included 142 long-chain ceramides and 38 ceramides; 225 *‘benzene and derivatives’* metabolites; 182 ‘*organooxygen compounds’* (including sugars and their derivatives); 164 ‘*sterol lipids*’ (ST) including 53 steroid esters; 42 ‘*bile acids*’ (BA); 153 ‘*prenol lipids’* (PR) that mostly consisted of 145 terpenoids; 99 ‘*glycerophospholipids*’ (GP) including 21 phosphatidylcholines (PC) and 14 phosphatidylethanolamines (PE); 70 ‘*organonitrogen compounds*’; and 50 ‘*pyridines and derivatives*’ (50). Other chemical classes with fewer than 50 compounds are given in Supplementary Table S1.

### 3.2. Quality Assessment of the Metabolome

To assess the precision of the overall analytical method, a quality control reference pool sample (QC) was constructed from all RGTMs to reflect an aggregated RGTMs metabolite composition. This pool QC was aliquoted and repeatedly injected between each set of 10 RGTMs samples. Overall precision was then evaluated via analysis of the total variance using principal component analysis (PCA). The PCA score plot in Figure S2 showed that the QC samples were aggregated into a tight cluster near the origin of the plot, indicating minimal residual technical errors. Conversely, metabolic data of the different fecal samples were scattered, explaining more than 73% of the total biological variance in the first two principal components. Using univariate analysis of all metabolites in the pool QC samples showed that nearly 71% of all annotated metabolites had excellent reproducibility with relative standard deviations (RSD) < 10%. A total of 89.2% of all metabolites were detected at RSD < 20%, highlighting the high quality of the data (Figure S3).

### 3.3. RGTM Assessment

The four RGTMs varied greatly in metabolite abundance due to the large differences in dietary habits of the study participants, and the impact of different RGTM preparations. Using PLS-DA multivariate analysis (Figure S4) and heatmap clustering (Figure S5), group-based differences were larger between the diets than between the lyophilized and aqueous sample pretreatments. To better understand the nature of these differences, we conducted a differentiated analysis per diet and per sample treatment.

### 3.4. Sample Treatment-Specific Compounds

**Figure 2:**
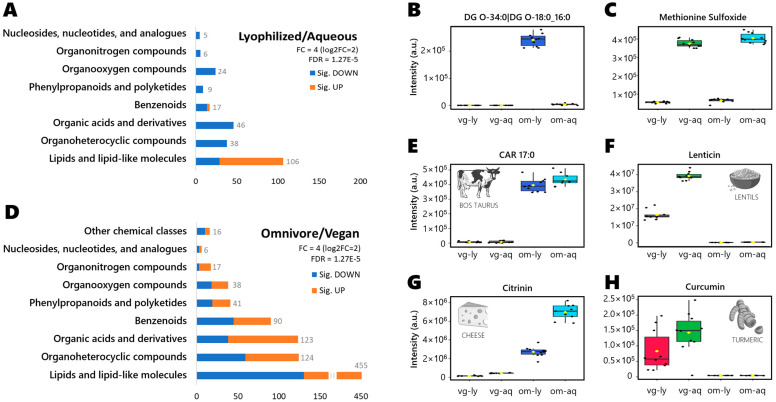
(**A**) Significant compounds grouped by chemical class per treatment at fold-change > 4 and Bonferroni FDR correction. Selected examples of distinctive compounds per sample group: (**B**) DG O-34 was only detected in lyophilized samples in omnivore diet. (**C**) Methionine sulfoxide detected at much higher levels in aqueous stool RGTM than in lyophilized samples. (**D**) Significant compounds grouped by chemical class per diet at fold-change > 4 and Bonferroni FDR correction. Selected examples of distinctive compounds per sample group: (**E**) Heptadecanoyl-carnitine (CAR 17:0) was only detected in omnivore samples, typically associated with animal-related foods (HMDB0006210, [19]). (**F**) Lenticin, a metabolite specific to lentils, found only in vegan stool samples. (**G**) Citrinin was found in omnivore samples, associated as fungi-specific metabolite from cheese fermentation processes [20]. (**H**) Spice metabolite from turmeric (curcumin), only detected in vegan samples.

Significantly altered compounds per treatment are shown in Figure 2A, grouped by chemical classes and with a minimum fold-change (FC) of four (log2FC = 2). Because 1001 compounds showed a raw significance of *p* < 0.05, we focused here on the true positives only, using a very conservative false discovery rate (FDR) restricted by Bonferroni correction. Of the 251 Bonferroni-significant compounds, almost half were assigned as lipids and lipid-like molecules, including 77 lipids that were more abundant in lyophilized samples and 29 lipids that were found in higher amounts in aqueous samples. Some examples of the compounds are shown in Figure 2B; the diglyceride DG O-34:0|DG O-18:0_16:0 was only found in omnivore lyophilized samples, while the amino acid derivate methionine sulfoxide was observed at higher intensity in aqueous samples (for both vegan and omnivores), as shown in Figure 2C.

### 3.5. Diet-Specific Metabolites

Significantly altered compounds per diet are shown in Figure 2D, grouped by chemical class per treatment at fold-change > 4 and Bonferroni FDR correction. Of the 910 compounds, half were classified as lipids and lipid-like molecules (325 lipids enriched in omnivore samples, 130 lipids more abundant in vegan samples). For example, heptadecanoyl carnitine or CAR 17:0 (Figure 2E) was only found in omnivores, as odd-chain acylcarnitines have been reported previously as biomarkers of dietary intake of fish or meat [21]. Similarly, lenticin (Figure 2F) is derived from the ingestion of lentils [22], explaining why we only found it in vegans. Other examples, could be citrinin (Figure 2G) found mainly in omnivore samples, as it derives from cheese production [20]; and curcumin (Figure 2H) found mainly in vegan samples, as it derives from turmeric ingestion [23]. Furthermore, the results obtained can be used to track the metabolic changes in specific pathways, like the purine metabolism pathway (Figure S6).

When specifically comparing published trends of metabolite differences between omnivores and vegans, the data presented here for NIST stool materials confirmed some reports but differed from others. For example, aspartate was higher in omnivores both in data reported here as well as in a recent multi-omics study [24]. Similarly, glucose and ribose levels were found at higher levels in omnivores than in vegans [25], validated by our data reported here. On the other hand, the same study [25] presented conflicting data with differences between NIST omnivore versus vegan stool. Unlike in the data we report here, in [25] hypoxanthine was found at higher levels in omnivores, not vegans. Conversely, urocanate, valine, leucine and isoleucine were previously reported at higher levels in vegetarians than in omnivores [25], which we could not confirm here.

**Figure 3:**
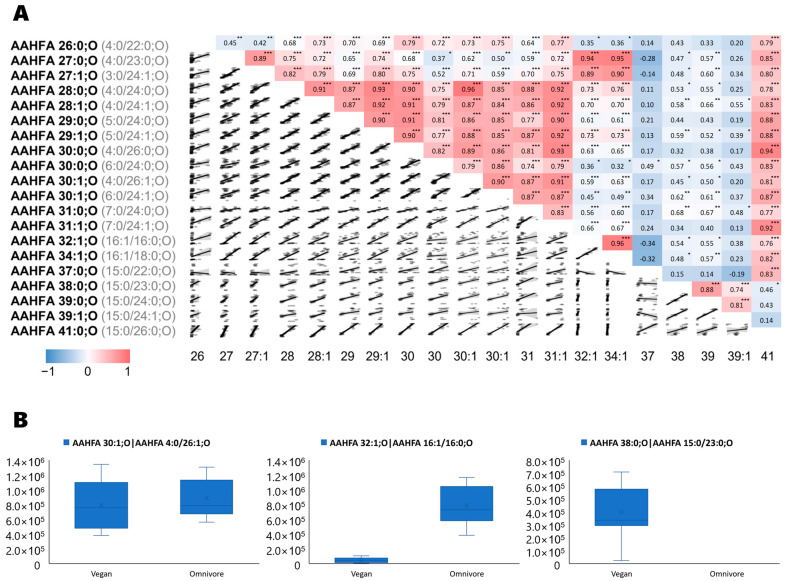
(**A**) Spearman correlations for each AAHFA lipid. Significance is indicated as * *p* < 0.05, ** *p* < 0.01 and *** *p* < 0.001. (**B**) Box-plots of examples of AAHFAs lipids. *Left panel:* AAHFA 30:1;O as example for AAHFA lipids detected in both diet-based fecal samples (AAHFA 26:0;O—AAHFA 31:1;O); *Mid panel:* AAHFA 32:1;O as example for lipids detected mostly in omnivore samples, e.g., also AAHFA 34:1;O); *Right panel:* AAHFA 38:0;O as example for lipids only found in vegans (AAHFA 37:0;O—AAHFA 41:0;O).

We also investigated microbiota-specific lipid structures like the acyl alpha-hydroxyl fatty acids (AAHFA) in their relationship to diet using Spearman-rank correlations (Figure 3A). We revealed three distinct patterns for the AHHFA chemical class of lipids, strongly suggesting that these compounds were produced by different microbial communities [26,27] that we found to be altered by diets. First, some AHHFAs were detected in both diet RGTM samples, such as AHHFA 30:1;O (Figure 3B). This type of compound correlated strongly with other mid-chain AHHFAs with less than 32 acyl-carbons (*r*_xy_ > 0.8, Figure 3A), but had a sharp drop in correlation strength for longer-chain AHHFAs. Second, some AHHFAs were found to be much more abundant in omnivore-derived fecal samples such as AAHHFA 32:1;O and AHHFA 34:1;O. The third group of AAHFAs was found in higher levels in vegan-derived fecal samples, including AAHFA 37:0;O, AHHFA 38:0;O, AAHFA 39:0;O, AAHFA 39:1;O, and AAHFA 41:0;O. Spectra and box-plots for each AAHFA are given in Supplementary Figure S7.

## 4. Conclusions

Reference materials (RMs) are critical for the successful development, validation, comparison, and standardization of clinical metabolomics assays. RMs are also needed as positive controls to monitor assay performance. This work provides a report of metabolome differences in four new human fecal RMs developed by the U.S. National Institute of Standards and Technologies (NIST) for metagenomics and metabolomics measurements. We used four untargeted metabolomics assays (lipidomics, primary metabolites, biogenic amines and polyphenols) and one targeted assay on bile acids. In total, 3563 compounds were annotated by mass spectrometry, including 353 compounds that were annotated in more than one assay. While many compounds were impacted by the sample pretreatment (lyophilized or aqueous), the overall magnitude of metabolic differences was larger when comparing fecal samples between dietary habits of subjects who donated fecal matter to the RGTM pools. In the fecal materials of vegans and omnivores, distinct regulation patterns were observed for bioactive molecules such as acyl alpha-hydroxy-fatty acids (AAHFA). This emphasizes the significance of using diet-specific reference materials in studies focused on dietary biomarkers.

## Data Availability

Datasets are available at the Zenodo repository (https://doi.org/10.5281/zenodo.7977475, accessed on 18 July 2023).

## Supplementary Materials

The following supporting information can be downloaded at: https://www.mdpi.com/article/10.3390/metabo13080921/s1, Figure S1: Fold-changes (FC) distribution by assay per vegan versus omnivore analysis; Figure S2: Fold-changes (FC) distribution by assay per vegan versus omnivore analysis; Figure S3: Distribution and cumulative percentage of technical variation of annotated metabolites in QC samples; Figure S4: PLS-DA with the z-score scale of the 4RGTMs (top left) with the loadings plot (middle) and some of the compound’s box-plots showing the differences in z-score from the different regions of the loadings plot. Data considered: CSH, HILIC, PFP, and GC; Figure S5: Heatmap of the 4RGTMs, with hierarchical clustering groups lyophilized and aqueous RGTMs; Figure S6: Reconstruction of the Purine metabolism pathway; Figure S7: AAHFA’s distribution per diet kind, and MSMS identification; Table S1: Untargeted results; Table S2: Targeted results.
