# Supplementary material for: Differences in the Stool Metabolome between Vegans and Omnivores: Analyzing the NIST Stool Reference Material
Source: Metabolites. 2023 Aug 7;13(8):921. doi: 10.3390/metabo13080921 (PMC10456543; doi:10.3390/metabo13080921)
Supplement: Supplementary file 1 [file metabolites-13-00921-s001.zip › Cumeras et al_NIST_stool_Metabolites_07-2023_supplement figures.pdf]

metabolites

Supplement information for

Differences in the Stool Metabolome between Vegans and Omnivores: Analyzing the NIST Stool Reference Material

Raquel Cumeras [1,2], Tong Shen [1], Luis Valdiviez [1], Zakery Tippins [1], Bennett D. Haffner [1] and Oliver Fiehn [1,*]

[1] West Coast Metabolomics Center, University of California Davis, Davis, CA 95616, USA; raquel.cumeras@urv.cat (R.C.); tsshen@ucdavis.edu (T.S.); lmvaldiviez@ucdavis.edu (L.V.); ztippins@ucdavis.edu (Z.T.); bdlikes3.14159@gmail.com (B.D.H.)

[2] Oncology Department, Nutrition and Metabolism Department, Institut d'Investigació Sanitària Pere Virgili (IISPV), Univeristat Rovira i Virgili (URV), 43204 Reus, Spain

* Correspondence: ofiehn@ucdavis.edu

Supplementary Figure S1. Fold-changes (FC) distribution by assay per vegan versus omnivore analysis.

Supplementary Figure S2. Fold-changes (FC) distribution by assay per vegan versus omnivore analysis.

Supplementary Figure S3. Distribution and cumulative percentage of technical variation of annotated metabolites in QC samples.

Supplementary Figure S4. PLS-DA with the z-score scale of the 4RGTMs (top left) with the loadings plot (middle) and some of the compound's boxplots showing the differences in z-score from the different regions of the loadings plot. Data considered: CSH, HILIC, PFP, and GC.

Supplementary Figure S5. Heatmap of the 4RGTMs, with hierarchical clustering groups lyophilized and aqueous RGTMs.

Supplementary Figure S6. Reconstruction of the Purine metabolism pathway.

Supplementary Figure S7. Distribution of AAHFAs per type of diet, and MSMS identification.

metabolites

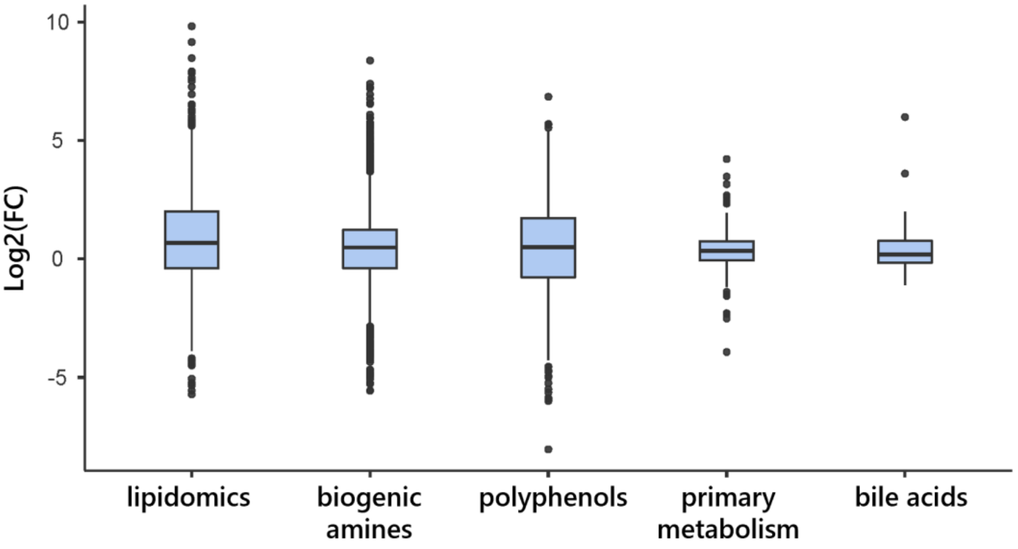

Figure S1. Fold-changes (FC) distribution by assay per vegan versus omnivore analysis.

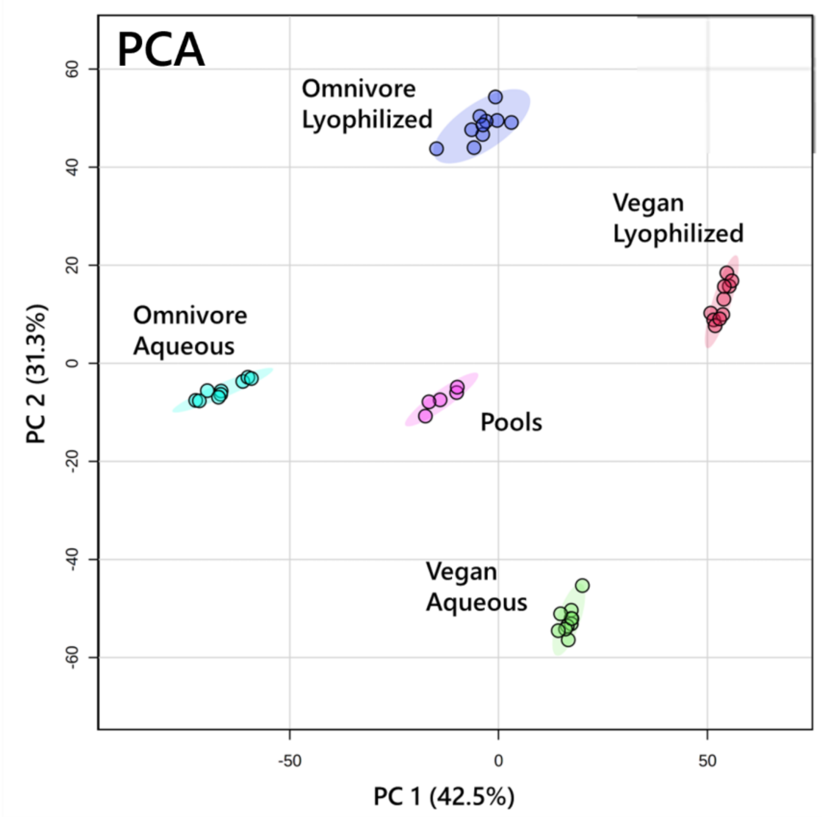

Figure S2. Fold-changes (FC) distribution by assay per vegan versus omnivore analysis.

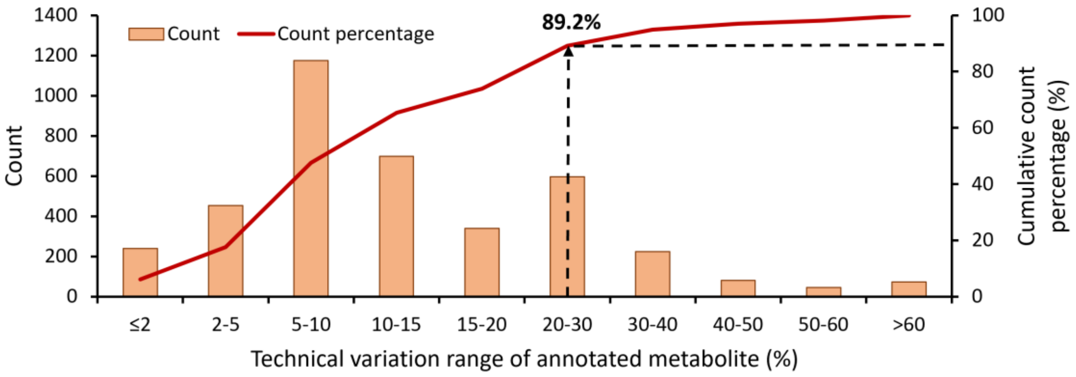

Figure S3. Distribution and cumulative percentage of technical variation of annotated metabolites in QC samples.

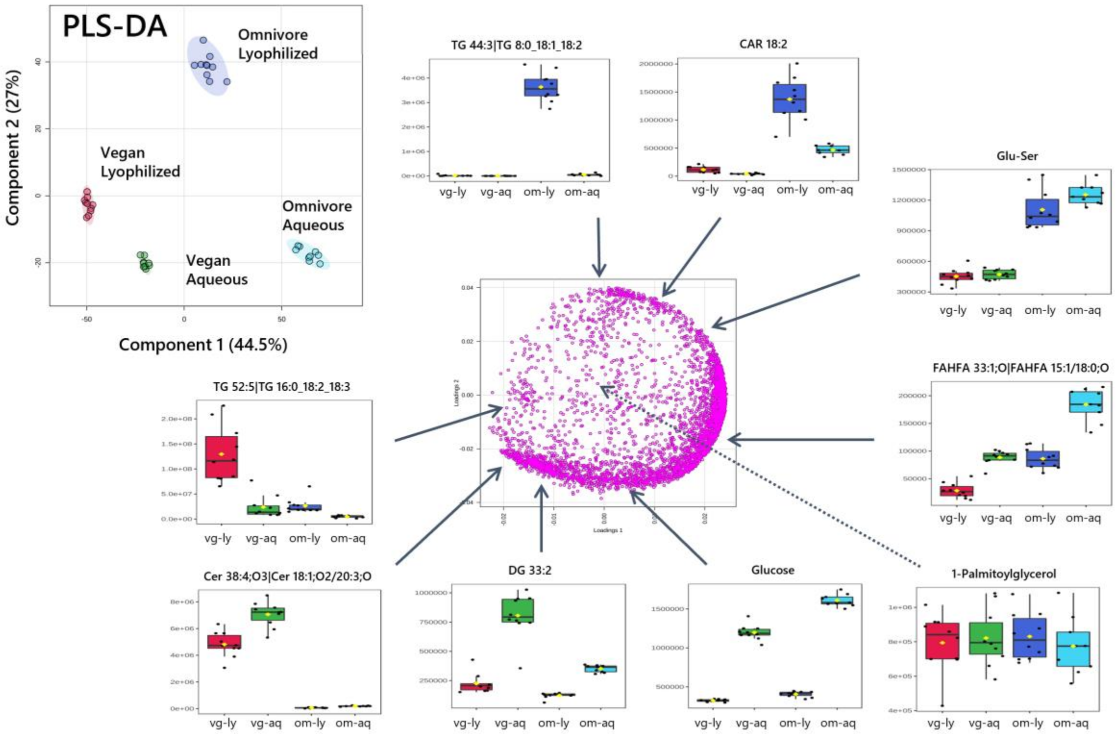

Figure S4. PLS-DA with the z-score scale of the 4RGTMs (top left) with the loadings plot (middle) and some of the compound’s boxplots showing the differences in z-score from the different regions of the loadings plot. Data considered: CSH, HILIC, PFP, and GC.

metabolites

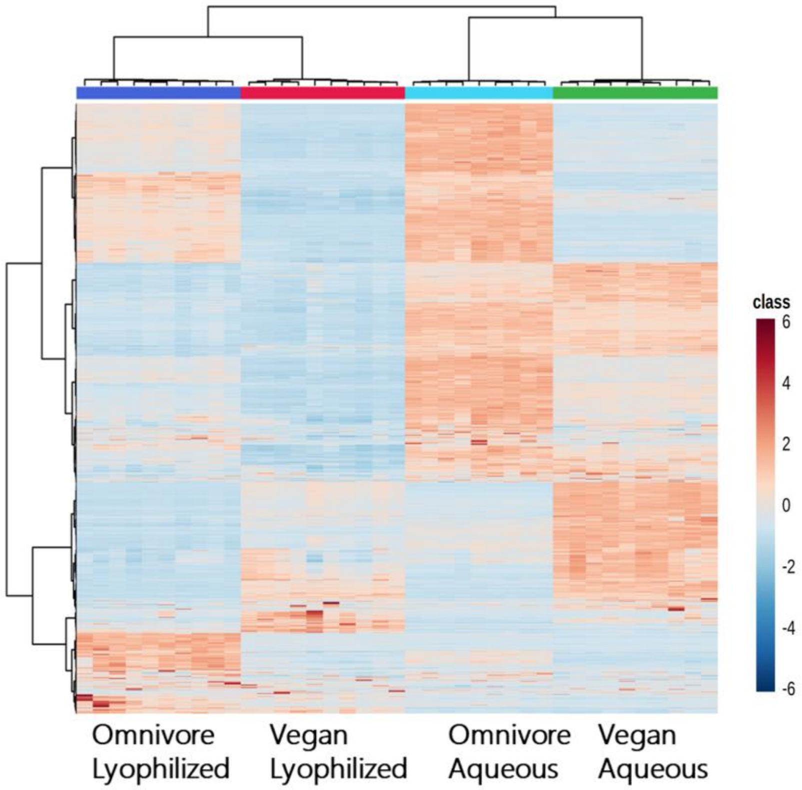

Figure S5. Heatmap of the 4RGTMs, with hierarchical clustering groups lyophilized and aqueous RGTMs.

metabolites

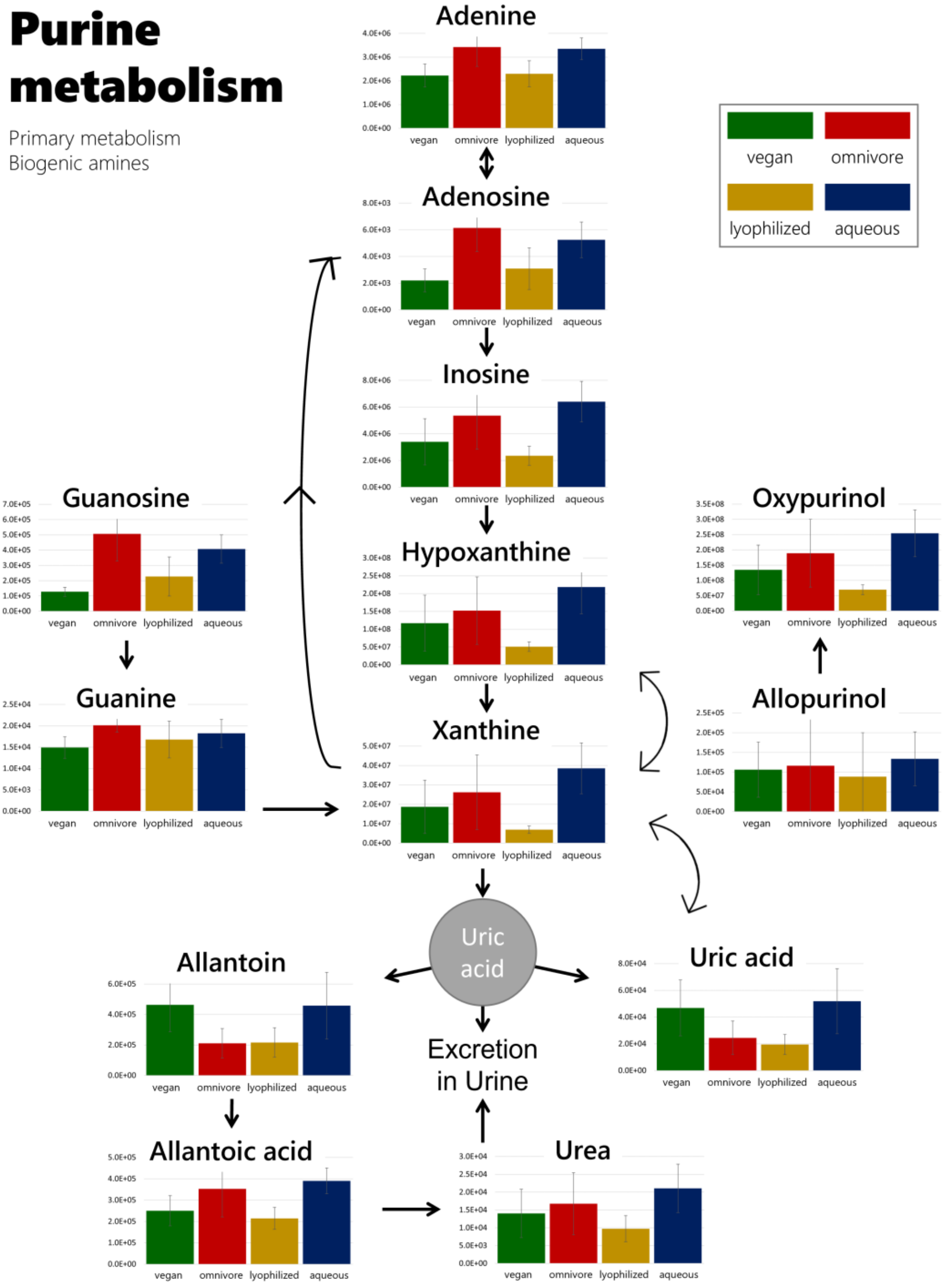

Figure S6. Reconstruction of the Purine metabolism pathway.

metabolites

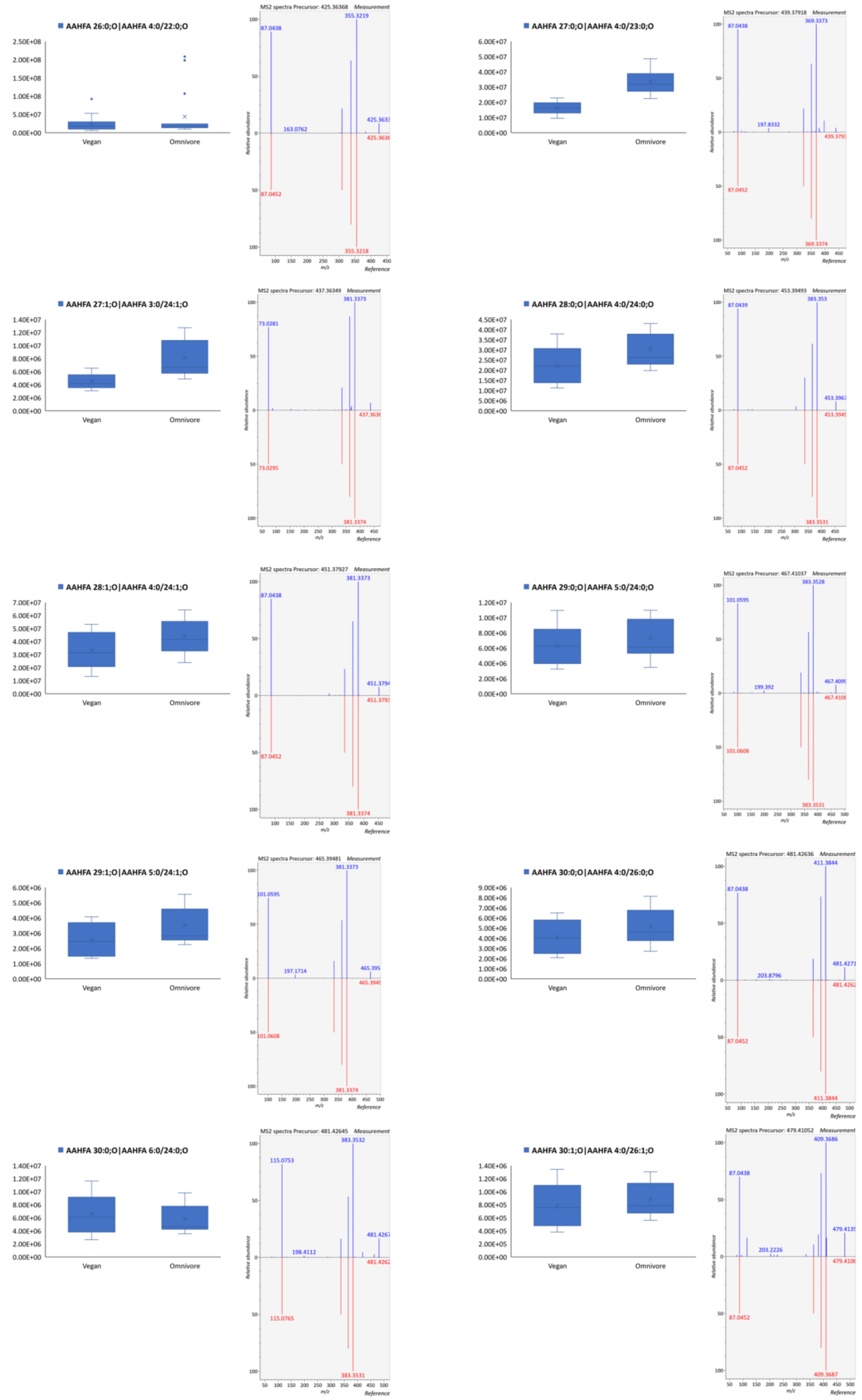

metabolites

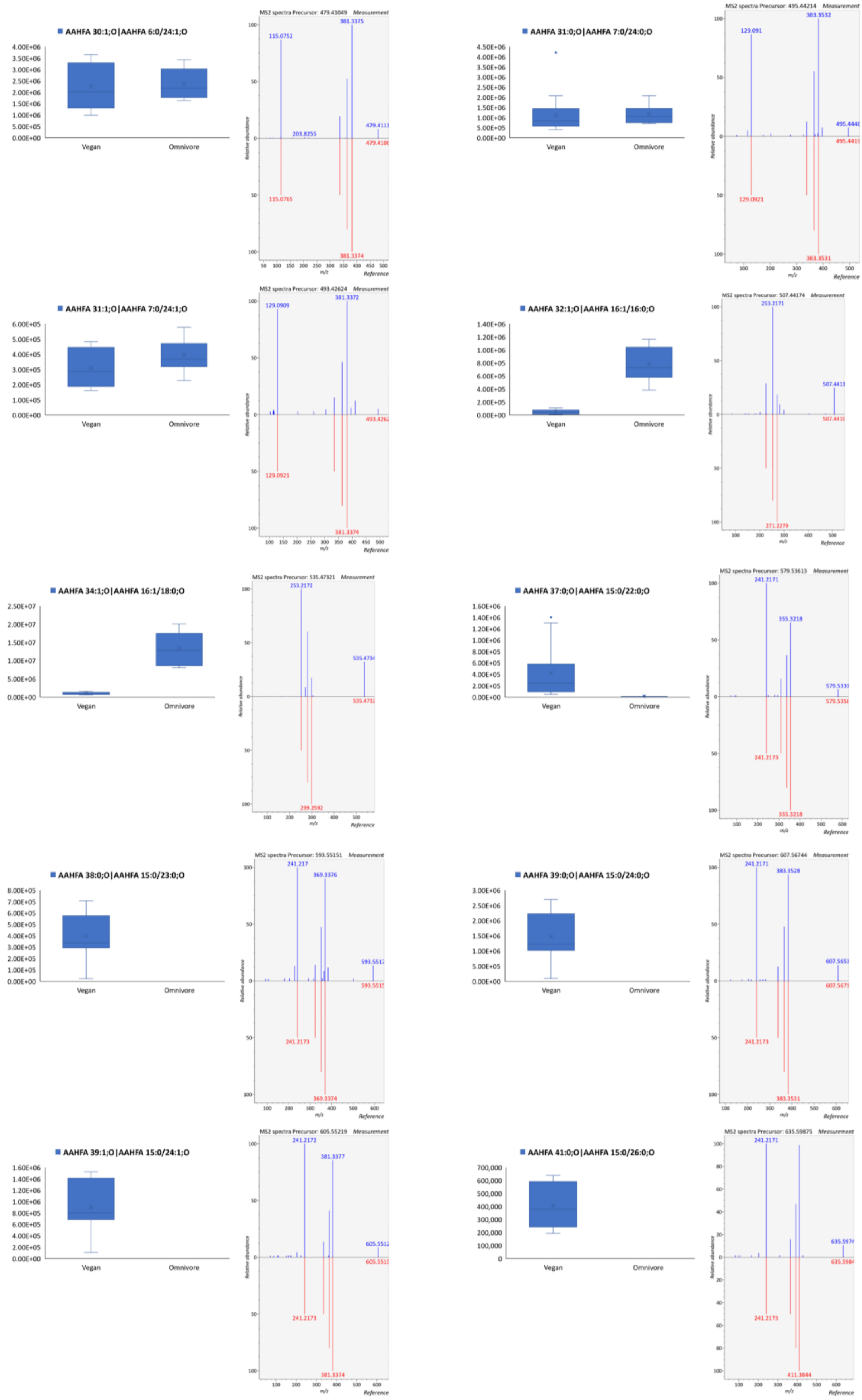

Figure S7. Distribution of AAHFAs per type of diet, and MSMS identification.
